# Small Bowel—Key Player in Health and Disease

**DOI:** 10.3390/jcm8101748

**Published:** 2019-10-21

**Authors:** Wojciech Marlicz, Anastasios Koulaouzidis

**Affiliations:** 1 Department of Gastroenterology, Pomeranian Medical University, 71-252 Szczecin, Poland; 2 Centre for Liver & Digestive Disorders, Royal Infirmary of Edinburgh, Edinburgh EH16 4SA, UK; akoulaouzidis@hotmail.com

Over the last two decades, remarkable progress has been made in understanding the etiology and pathophysiology of diseases. New discoveries emphasize the importance of the small bowel (SB) ‘ecosystem’ in the pathogenesis of acute and chronic illness alike. Emerging factors, such as microbiome, stem and progenitor cells, innate intestinal immunity, and the enteric nervous system, along with mucosal and endothelial barriers, play a key role in the development of gastrointestinal (GI) and extra-GI diseases. The results of other studies point also towards a link between the digestive tract and common non-communicable diseases, such as obesity and cancer. These discoveries unravel novel dimensions of uncertainty in the area of clinical decision-making motivating researchers to search for novel diagnostic and therapeutic solutions. 

Recent studies unravel the role of the poorly-understood complexity of the SB. Insights into its critical physiologic and pathophysiologic role in metabolic homeostasis and its potential role as a driver of obesity, insulin resistance, and subsequent type 2 diabetes mellitus (T2DM) have been revealed [1]. For example, bypassing the proximal small intestine by means of bariatric surgery results in a significant metabolic benefit to an individual undergoing such a procedure. Moreover, endoscopic procedures aimed at placing devices separating luminal contents from the duodenal mucosa result in modest weight loss and improvement in glucose homeostasis [1]. 

Another endoscopic treatment which aims at resurfacing duodenal mucosa (DMR, duodenal mucosal resurfacing), leads to improvement in glycemia and insulin resistance in patients with T2DM [2]. It is not surprising when we recall that SB endocrine cells secrete glycemia-regulating incretin hormones (e.g., glucagon-like peptide, GLP-1), and this process is dependent on food content in the intestinal lumen. Moreover, bile acids with various targets in the liver and small intestine (e.g., farnesoid receptor, FXR) together with a plethora of other small signaling molecules act in concert in regulating metabolic and digestive GI function.

The human digestive tract and enteric nervous system (ENS) communicate with the central nervous system (CNS) through the gut-brain axis (GBA). This bidirectional communication involves diverse neural networks through the X cranial vagal nerve—dorsal roots of the sympathetic/ parasympathetic nervous system. Important roles in the regulation of the gut-brain axis are played by: (i) the hypothalamus-pituitary-adrenal axis (HPA), (ii) the stress hormones (cortisol), (iii) the short-chain fatty acids (SCFAs), and (iv) the gut microbiota. The intestinal barrier, another important part of GBA, is composed of: (i) goblet cells derived mucus, (ii) microbiota, (iii) epithelial cells, (iv) endothelial cells, (v) lymphatic vessels, and v) enterocytes’ tight cellular junctions. Of interest, the structure and function of the intestinal barrier resemble that of the blood-brain barrier (BBB). The gut-brain communication is mediated via blood, portal/hepatic circulation, and the bone marrow [3]. 

Recently, it has been described that the alterations of the intestinal barrier play a crucial role in the pathology of several human inflammatory and autoimmune diseases. Mohanan et al. documented the crucial role of the C1orf106 inflammatory bowel disease (IBD) susceptibility gene in stabilizing intestinal barrier function and intestinal inflammation [4]. Manfredo Vieira et al. evidenced the role of pathobionts in the process of intestinal barrier alterations, which were followed by their translocation to lymph nodes and the hepatic portal system, triggering systemic lupus erythematosus (SLE) [5]. Thaiss et al. reported that hyperglycemia leads to disruption of the intestinal barrier followed by intestinal inflammation and systemic infection [6]. Spadoni et al. recently documented that the presence of the gut-vascular barrier (GVB) in the small intestine controls the dissemination of bacteria into the bloodstream [7]. The authors reported a decrease of the wnt/beta catenin-inducible gene Axin2 (a marker of stem cell renewal) in gut endothelium under the presence of *Salmonella typhimirum* in the SB. The GVB was modified in patients with coeliac disease (CD) with altered serum transaminases, which suggests that GVB deterioration may be responsible for liver damage in CD patients [7]. It has been shown that disruption of epithelial and vascular barriers in the intestine were early events in non-alcoholic steatohepatitis (NASH), and GVB leakage marker could be identified in colonic biopsies in patients with NASH [8]. Of importance, SB epithelial and vascular barriers are FXR-controlled, which opens avenues to clinical trials aimed at investigation of novel FXR-agonists as future therapeutics [9]. Of interest, intestinal barriers could be monitored in vivo with the aid of confocal laser endomicroscopy (CLE) [10,11]. 

Therefore, the SB is considered a key player in metabolic disease development [12], including diabetes mellitus and NASH, and other diet-related disorders such as coeliac and non-coeliac enteropathies. Another major field is drug metabolism and its interaction with small bowel microbiome [13]. Moreover, the emergence of gut-brain, gut-liver, and gut-blood barriers point towards the important role of the SB in the pathogenesis of previously unthought and GI-unrelated conditions such as neurodegenerative and cardiovascular disease [14,15]. The SB remains an organ that is difficult to fully access and assess and accurate diagnosis often poses a clinical challenge. Undoubtedly, the therapeutic potential remains untapped. Therefore, it is now time to direct more of our interest towards the SB and unravel the interplay between the SB and other GI and non-GI related diseases. 

In this Journal of Clinical Medicine Special Issue, “Diagnosis and Treatment of Small Bowel Disorders”, several groups of investigators contributed their knowledge to the field of SB research by presenting original papers and reviews. 

Enaud et al. [16] describe original observations by utilizing next-generation sequencing (NGS), that intestinal inflammation in children with Cystic Fibrosis (CF) was associated with alterations of microbiota similar to those observed in Crohn’s disease (CrDs). Authors for the first time applied novel CrDs Microbial-Dysbiosis index in CF patients and pointed towards the importance of gut-lung axis in CF prognosis [16]. Of interest, intestinal inflammation was associated with previous intravenous antibiotic courses for CF [13]. This observation is important as global awareness of antibiotic resistance rises. Nakamura et al. [17] sought to evaluate the validity of using capsule endoscopy (CE) to monitor the effect of medical treatment on SB mucosal healing in post-operative CrDs patients, regardless of the presence of clinical symptoms. Although the significance of endoscopic monitoring has been widely accepted in CrDs, to date, only a few studies looked at the validity and effectiveness of escalating treatment for patients in clinical remission but with endoscopically visible active mucosal lesion. The authors demonstrated that CrDs patients in clinical remission with ongoing intestinal inflammation at the time of the CE could benefit from additional treatment. This study motivates physicians to optimize treatment plans for asymptomatic CD patients.

Several characteristics of SB lesions (e.g., mucosal disruption, bleeding, irregular surface, polypoid appearance, color, delayed passage, white villi, and invagination) have been described to better predict SB lesions, such as intestinal bulges, masses, and tumors. The lack of precise features in characterizing these lesions places a limitation on the accuracy of CE diagnosis. Therefore, Min et al. [18] in their retrospective study, evaluated the utility of an additional morphologic criterion, the mucosal protrusion angle (MPA), which was defined as the angle between a SB protruding lesion and its surrounding mucosa. The authors documented that MPA was a simple and useful tool for differentiating between intestinal true masses and non-significant bulges [18]. Their observation creates a useful extra tool for those who are faced with the question of ‘mass or bulge?’ 

SB microbiota alterations have also been implicated in the pathogenesis of surgical site infections (SSIs) and surgery-related complications (SRCs). Skonieczna-Żydecka et al. [19] conducted a systematic review with meta-analysis and meta-regression of randomized clinical trials investigating the efficacy of probiotics and synbiotics to counteract SSIs and SRCs in patients under various surgical treatments. The authors aimed to determine the mechanisms behind probiotic/synbiotic action. Their meta-analysis revealed that probiotics/synbiotics administration prior and at a time of major abdominal surgery, leads to a reduction in the incidence of SSIs and SRCs (e.g., abdominal distension, diarrhea, pneumonia, sepsis, urinary tract infection, postoperative pyrexia). Furthermore, probiotics/synbiotics were associated with shortening of the duration of antibiotic therapy and hospital stay. Based on current evidence, the action of probiotics/synbiotics in surgical patients seems to be exerted via modulation of gut-immune response and production of short-chain fatty acids (SCFAs) [19].

Included also in this special issue, Singh et al. [20] comprehensively reviewed the pros and cons of biomarkers in coeliac disease and summarized the current status of coeliac disease screening, diagnosis, and monitoring. The review could guide clinicians in diagnosis and monitoring of patients with coeliac disease. As biomarkers allow for smart targeted-screening, similarly imaging spectroscopy (a combination of digital imaging and spectroscopy, also known as hyperspectral/multispectral (HS/MS) imaging (HSI/MSI) technology) allows for smart tissue visualization beyond the limitations of the human eye. HSI has been utilized for various research purposes including: (i) food quality inspection, (ii) optimization of the recycling process, (iii) art painting renovations, (iv) geology and minerals inspection, (v) soil evaluation and (vi) plant response to stress. HSI has also been evolving in the field of medical research in gastroenterology, pathology, and surgery. This modality was used to generate alternative visualization of tissues, abdominal organ differentiation, identification of surgical site resection, and abdominal ischemia to name a few. Ortega et al. [21] in their thorough review provided a detailed summary of the most relevant research work in the field of gastroenterology using HSI.

Last but not least, Skonieczna-Żydecka et al. [22] in a narrative review published in this issue of Journal of Clinical Medicine, discussed involvements of GBA deregulation in the origin of brain-gut disorders. The authors hypothesized that stem cell-host microbiome cross-talk was potentially involved in GBA disorders. Interestingly, patients with inflammatory bowel disease (IBD) have an elevated risk of mental illness, and depression increases the risk of IBD. Of key interest, are observations that multiple drugs are known to induce metabolic malfunctions, possibly through alterations of SB milieu. These alterations result in body weight gain, metabolic disturbances, and suppression of metabolic resting rate. The authors in their comprehensive review presented the current state of the art knowledge of the role of GBA in GI and psychiatric comorbidities. The current evidence supports the notion that an injury to the intestinal mucosa can result in significant, though delayed, metabolic consequences that may seriously affect the health of an individual. Future investigations [23] into the pathophysiology of host-microbe interactions should focus on the small bowel [24,25], which is still relatively inaccessible. Therefore, the research is challenging but necessary to pave the way to new findings and solutions in the clinical area of the small bowel [26] and beyond [27].

Dr Marlicz and Dr Koulaouzidis are Guest Editors of this Special Issue of the Journal of Clinical Medicine (ISSN 2077-03830), which belongs to the journal’s section Gastroenterology and Hepato-Pancreato-Biliary Medicine.

## References

[1] Van Baar A.C.G., Nieuwdorp M., Holleman F., Soeters M.R., Groen A.K., Bergman J.J.G.H.M. (2018). The Duodenum harbors a Broad Untapped Therapeutic Potential. Gastroenterology.

[2] Van Baar A.C.G., Holleman F., Crenier L., Haidry R., Magee C., Hopkins D., Rodriguez Grunert L., Galvao Neto M., Vignolo P., Hayee B. (2019). Duodenal mucosal resurfacing for the treatment of type 2 diabetes mellitus: One year results from the first international, open-label, prospective, multicentre study. Gut.

[3] Odenwald M.A., Turner J.R. (2017). The intestinal epithelial barrier: A therapeutic target?. Nat. Rev. Gastroenterol. Hepatol..

[4] Mohanan V., Nakata T., Desch A.N., Lévesque C., Boroughs A., Guzman G., Cao Z., Creasey E., Yao J., Boucher G. (2018). *C1orf106* is a colitis risk gene that regulates stability of epithelial adherens junctions. Science.

[5] Vieira S.M., Hiltensperger M., Kumar V., Zegarra-Ruiz D., Dehner C., Khan N., Costa F.R.C., Tiniakou E., Greiling T., Ruff W. (2018). Translocation of a gut pathobiont drives autoimmunity in mice and humans. Science.

[6] Thaiss C.A., Levy M., Grosheva I., Zheng D., Soffer E., Blacher E., Braverman S., Tengeler A.C., Barak O., Elazar M. (2018). Hyperglycemia drives intestinal barrier dysfunction and risk for enteric infection. Science.

[7] Spadoni I., Zagato E., Bertocchi A., Paolinelli R., Hot E., Di Sabatino A., Caprioli F., Bottiglieri L., Oldani A., Viale G. (2015). A gut-vascular barrier controls the systemic dissemination of bacteria. Science.

[8] Mouries J., Brescia P., Silvestri A., Spadoni I., Sorribas M., Wiest R., Mileti E., Galbiati M., Invernizzi P., Adorini L. (2019). Microbiota-driven gut vascular barrier disruption is a prerequisite for non-alcoholic steatohepatitis development. J. Hepatol..

[9] Neuschwander-Tetri B.A., Loomba R., Sanyal A.J., Lavine J.E., Van Natta M.L., Abdelmalek M.F., Chalasani N., Dasarathy S., Diehl A.M., Hameed B. (2015). NASH Clinical Research Network. Farnesoid X nuclear receptor ligand obeticholic acid for non-cirrhotic, non-alcoholic steatohepatitis (FLINT): A multicentre, randomised, placebo-controlled trial. Lancet.

[10] Sorribas M., Jakob M.O., Yilmaz B., Li H., Stutz D., Noser Y., de Gottardi A., Moghadamrad S., Hassan M., Albillos A. (2019). FXR-modulates the gut-vascular barrier by regulating the entry sites for bacterial translocation in experimental cirrhosis. J. Hepatol..

[11] Fritscher-Ravens A., Pflaum T., Mösinger M., Ruchay Z., Röcken C., Milla P.J., Das M., Böttner M., Wedel T., Schuppan D. (2019). Many Patients with Irritable Bowel Syndrome Have Atypical Food Allergies Not Associated with Immunoglobulin E. Gastroenterology.

[12] Tilg H., Zmora N., Adolph T.E., Elinav E. (2019). The intestinal microbiota fuelling metabolic inflammation. Nat. Rev. Immunol..

[13] Skonieczna-Żydecka K., Łoniewski I., Misera A., Stachowska E., Maciejewska D., Marlicz W., Galling B. (2019). Second-generation antipsychotics and metabolism alterations: A systematic review of the role of the gut microbiome. Psychopharmacology.

[14] Tanaka M., Itoh H. (2019). Hypertension as a Metabolic Disorder and the Novel Role of the Gut. Curr. Hypertens Rep..

[15] Kowalski K., Mulak A. (2019). Brain-Gut-Microbiota Axis in Alzheimer’s Disease. J. Neurogastroenterol. Motil..

[16] Enaud R., Hooks K.B., Barre A., Barnetche T., Hubert C., Massot M., Bazin T., Clouzeau H., Bui S., Fayon M. (2019). Intestinal Inflammation in Children with Cystic Fibrosis Is Associated with Crohn’s-Like Microbiota Disturbances. J. Clin. Med..

[17] Nakamura M., Yamamura T., Maeda K., Sawada T., Mizutani Y., Ishikawa T., Furukawa K., Ohno E., Kawashima H., Miyahara R. (2018). Nagoya University Crohn’s Disease Study Group. Validity of Capsule Endoscopy in Monitoring Therapeutic Interventions in Patients with Crohn’s Disease. J. Clin. Med..

[18] Min M., Noujaim M.G., Green J., Schlieve C.R., Vaze A., Cahan M.A., Cave D.R. (2019). Role of Mucosal Protrusion Angle in Discriminating between True and False Masses of the Small Bowel on Video Capsule Endoscopy. J. Clin. Med..

[19] Skonieczna-Żydecka K., Kaczmarczyk M., Łoniewski I., Lara L.F., Koulaouzidis A., Misera A., Maciejewska D., Marlicz W. (2018). A Systematic Review, Meta-Analysis, and Meta-Regression Evaluating the Efficacy and Mechanisms of Action of Probiotics and Synbiotics in the Prevention of Surgical Site Infections and Surgery-Related Complications. J. Clin. Med..

[20] Singh A., Pramanik A., Acharya P., Makharia G.K. (2019). Non-Invasive Biomarkers for Celiac Disease. J. Clin. Med..

[21] Ortega S., Fabelo H., Iakovidis D.K., Koulaouzidis A., Callico G.M. (2019). Use of Hyperspectral/Multispectral Imaging in Gastroenterology. Shedding Some Different Light into the Dark. J. Clin. Med..

[22] Skonieczna-Żydecka K., Marlicz W., Misera A., Koulaouzidis A., Łoniewski I. (2018). Microbiome—The Missing Link in the Gut-Brain Axis: Focus on Its Role in Gastrointestinal and Mental Health. J. Clin. Med..

[23] Pełka-Wysiecka J., Kaczmarczyk M., Bąba-Kubiś A., Liśkiewicz P., Wroński M., Skonieczna-Żydecka K., Marlicz W., Misiak B., Starzyńska T., Kucharska-Mazur J. (2019). Analysis of Gut Microbiota and Their Metabolic Potential in Patients with Schizophrenia Treated with Olanzapine: Results from a Six-Week Observational Prospective Cohort Study. J. Clin. Med..

[24] Zhong L., Shanahan E.R., Raj A., Koloski N.A., Fletcher L., Morrison M., Walker M.M., Talley N.J., Holtmann G. (2017). Dyspepsia and the microbiome: Time to focus on the small intestine. Gut.

[25] Vuik F., Dicksved J., Lam S.Y., Fuhler G.M., van der Laan L., van de Winkel A., Konstantinov S.R., Spaander M., Peppelenbosch M.P., Engstrand L. (2019). Composition of the mucosa-associated microbiota along the entire gastrointestinal tract of human individuals. United Eur. Gastroenterol. J..

[26] Quigley E.M.M. (2019). Symptoms and the small intestinal microbiome—The unknown explored. Nat. Rev. Gastroenterol. Hepatol..

[27] Lynch S.V., Ng S.C., Shanahan F., Tilg H. (2019). Translating the gut microbiome: Ready for the clinic?. Nat. Rev. Gastroenterol. Hepatol..

